# Supplementary figures and images for: Identification of the cleavage sites leading to the shed forms of human and mouse anti-aging and cognition-enhancing protein Klotho
Source: PLoS One. 2020 Jan 13;15(1):e0226382. doi: 10.1371/journal.pone.0226382 (PMC6957300; doi:10.1371/journal.pone.0226382)

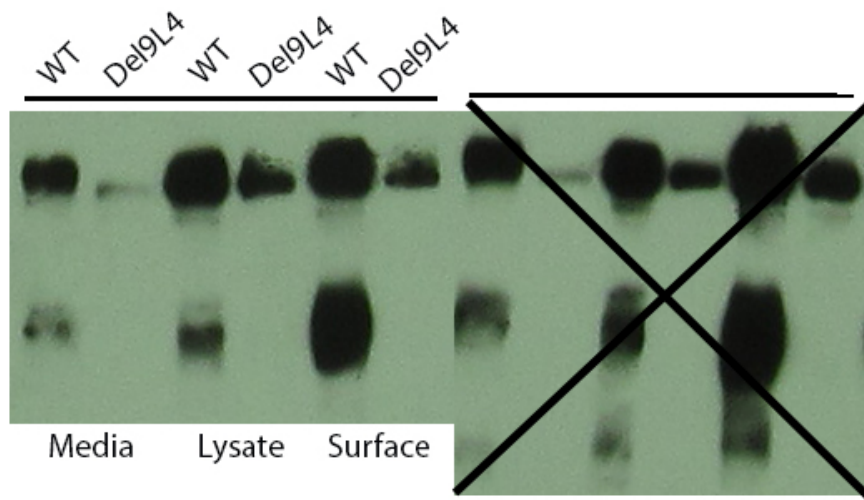

**S1A Figure**

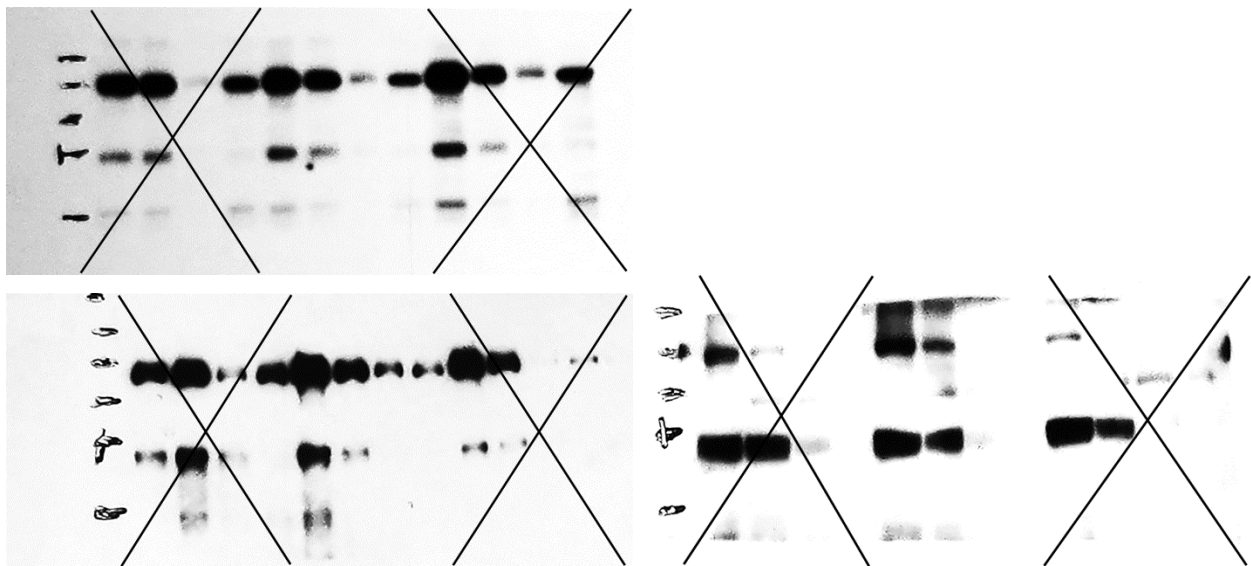

**S1B Figure**

Supplement: S1 Fig — S1A The figure on the left was used to generate Fig 2A. S1B The middle figures were used to generate Fig 2B. (PDF) [file pone.0226382.s001.pdf]

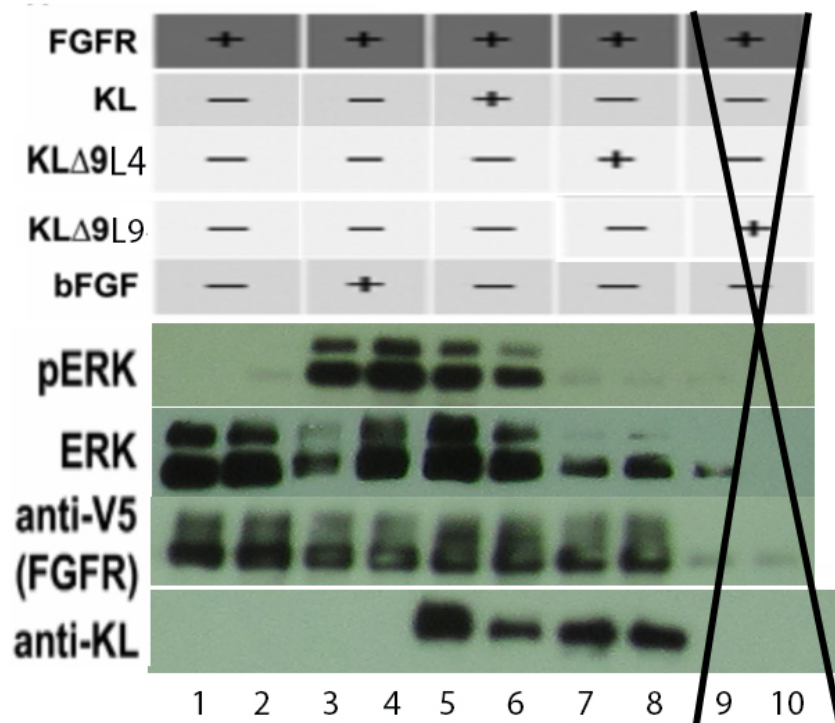

S2A Figure

a

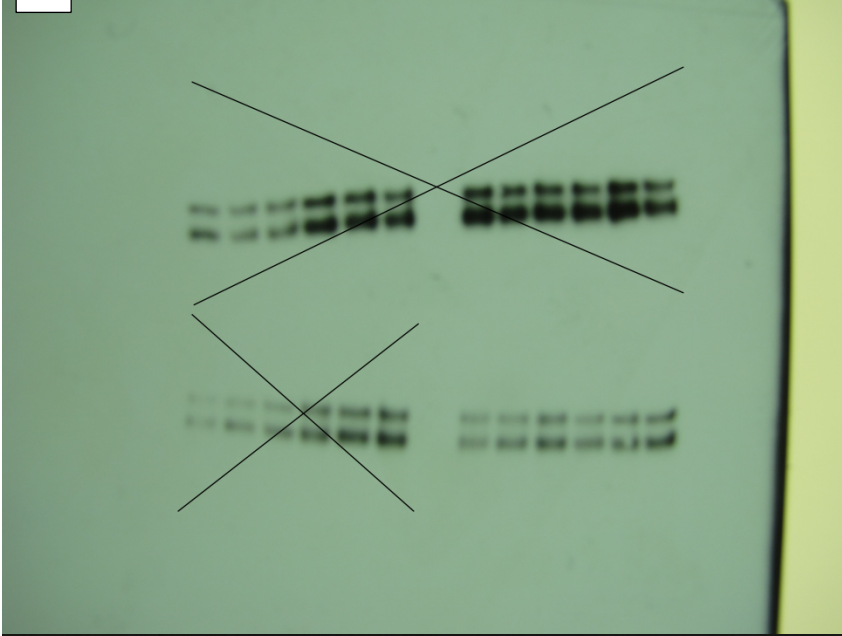

b

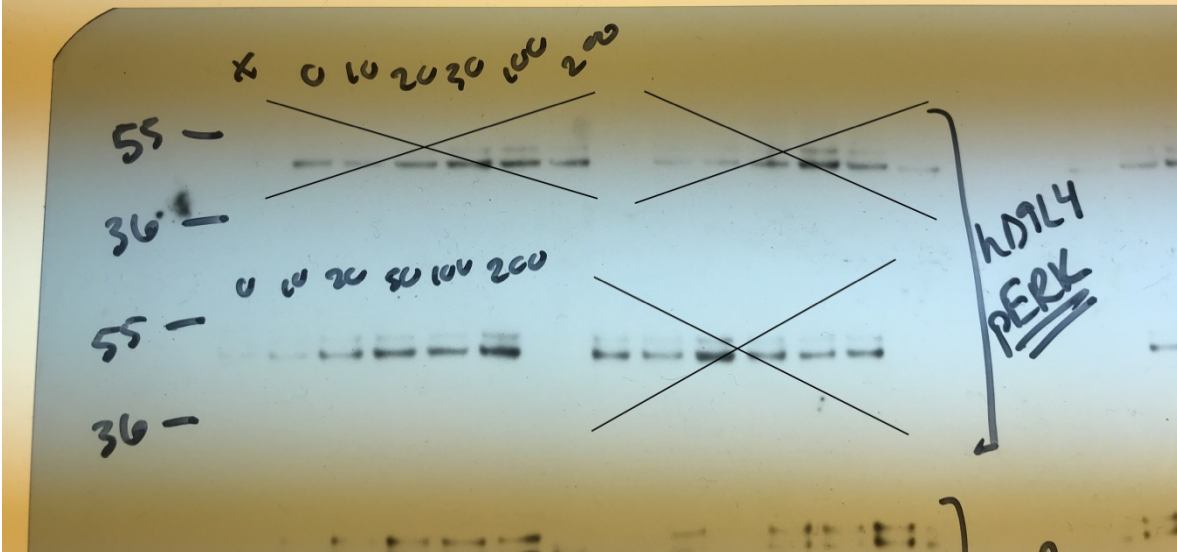

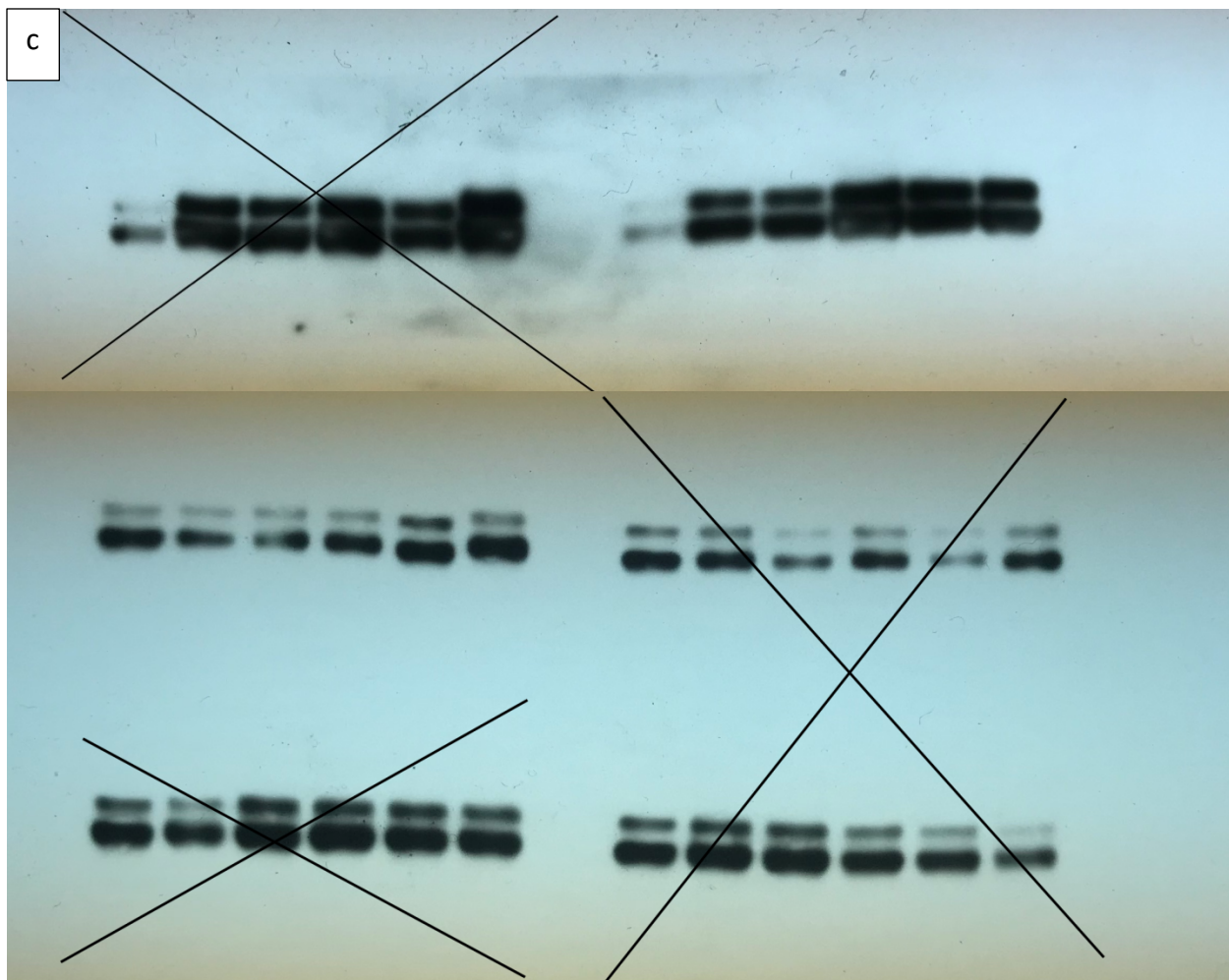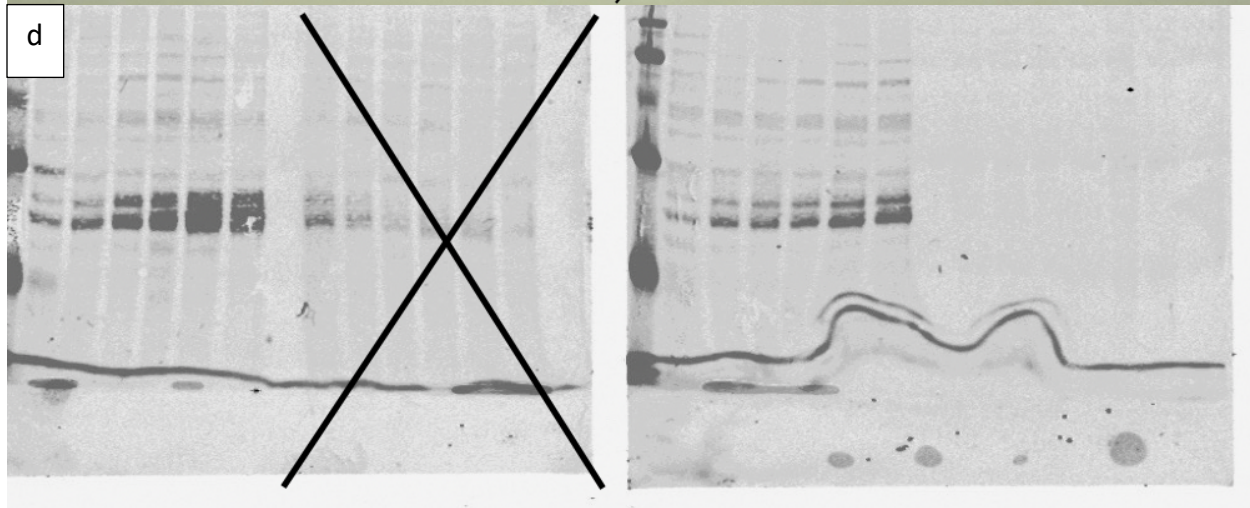

e

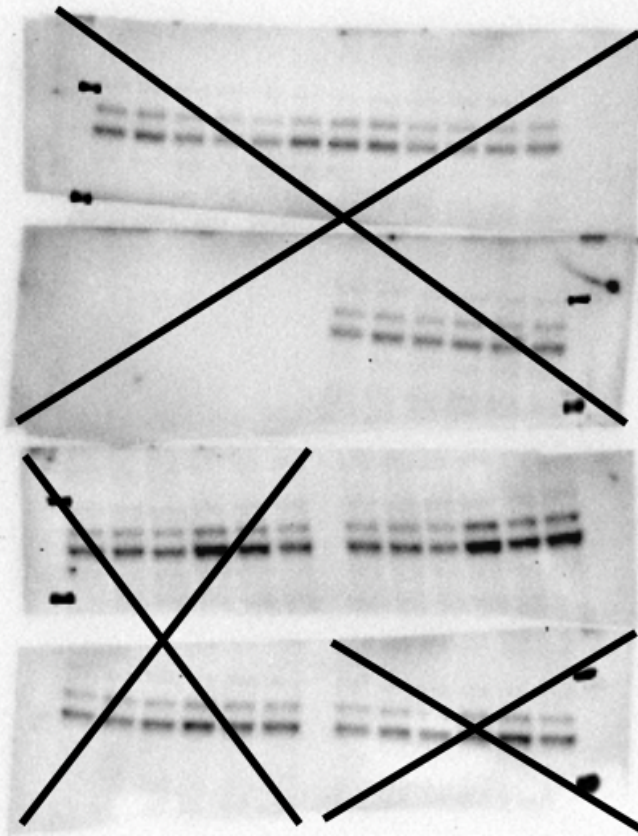

S2B Fig

Supplement: S2 Fig — S2A image was used to generate Fig 4A. S2B a, b, c. pERK and ERK blots for hKL. Only one of the repeats was used for the publication. S2B d, e The images were used for pERK for mKL and mKLD9. For all other ERK blots, the uncropped images were not saved because the cropped blot had the only visible bands. (PDF) [file pone.0226382.s002.pdf]
