# Supplementary material for: Identification of the cleavage sites leading to the shed forms of human and mouse anti-aging and cognition-enhancing protein Klotho
Source: PLoS One. 2020 Jan 13;15(1):e0226382. doi: 10.1371/journal.pone.0226382 (PMC6957300; doi:10.1371/journal.pone.0226382)
Supplement: Attachment — Submitted filename: Response to reviewers.docx [file pone.0226382.s003.docx]

**Response to reviewers**

We are very grateful to the reviewer for the suggestions to improve our manuscript. Our responses are listed below. We hope the new revised manuscript is now suitable for publication in PLOS ONE. Thank you for considering our submission.

Reviewer #1: The authors present an interesting study with the aim of identifying cleavage sites leading to the shed forms of human and mouse Klotho protein. It is largely a well written manuscript which is clearly laid out. I have a few minor comments as below:

1. A reference has been missed in the introduction, paragraph 2 "Mice deficient in Klotho exhibit changes observed during human ageing"

***Response:*** The reference has been added.

2. "They also have a shortened life span"

***Response:*** The word “shortened” has been corrected.

3. The figure legends are usually at the end of the paper

***Response:*** The figure legends are now moved to the end of the paper.

In addition, we provided in Supporting Information a document with the non-cropped western blots shown.

Thank you,

The authors
